# Contrasting Nitrogen Fertilisation Rates Alter Mycorrhizal Contribution to Barley Nutrition in a Field Trial

**DOI:** 10.3389/fpls.2019.01312

**Published:** 2019-10-30

**Authors:** Tom Thirkell, Duncan Cameron, Angela Hodge

**Affiliations:** ^1^Department of Biology, University of York, York, United Kingdom; ^2^Department of Animal and Plant Sciences, University of Sheffield, Sheffield, United Kingdom

**Keywords:** arbuscular mycorrhiza, nitrogen, barley, field trial, plant ecophysiology

## Abstract

Controlled environment studies show that arbuscular mycorrhizal fungi (AMF) may contribute to plant nitrogen (N) uptake, but the role of these near-ubiquitous symbionts in crop plant N nutrition under natural field conditions remains largely unknown. In a field trial, we tested the effects of N fertilisation and barley (*Hordeum vulgare* L.) cultivar identity on the contribution of AMF to barley N uptake using ^15^N tracers added to rhizosphere soil compartments. AMF were shown capable of significantly increasing plant ^15^N acquisition from root exclusion zones, and this was influenced by nitrogen addition type, N fertiliser application rate and barley cultivar identity. Our data demonstrate a previously overlooked potential route of crop plant N uptake which may be influenced substantially and rapidly in response to shifting agricultural management practices.

## Introduction

Nitrogen (N) is usually the most limiting mineral nutrient to plant growth (Agren et al., 2012) and maintaining modern agricultural production requires frequent and substantial application of fertiliser to farm soils. In various forms an estimated 50 MT year^−1^ fertiliser N is applied to agricultural land worldwide (Ladha et al., 2016). Assimilation of applied N by crops may be under 50% (Ladha et al., 2005, Masclaux-Daubresse et al., 2010); a significant fraction of this applied N is wasted — lost through processes including volatilisation, microbial immobilisation, runoff, and leaching (Ladha et al., 2016, Cameron et al., 2013). There is economic and ecological pressure on farmers to optimise the N uptake efficiency of crop plants (Hawkesford, 2014) and by reducing the reliance on non-renewable inputs, improve the sustainability of agriculture (Pretty, 2008). This progress will require the integration of biological and ecological processes into agriculture, and better understanding of soil microbial communities and their roles in nutrient cycling (Rillig et al., 2016, Pretty, 2018).

As near-ubiquitous symbionts of cereal crops, arbuscular mycorrhizal fungi (AMF) are prime targets to investigate the role of soil biota in improving agricultural sustainability (Gosling et al., 2006, Thirkell et al., 2017, Rillig et al., 2019). The majority of land plant species engage in symbiosis with these fungi, which may aid plants’ mineral nutrient uptake from soils, in exchange for photosynthetic carbon (C) from their plant hosts (Smith and Read, 2008). The influence that AMF mycelia may exert over nutrient dynamics in agricultural systems is not limited to direct effects on plant nutrient acquisition however; the presence of AMF has been shown to reduce mineral fertiliser leaching (Cavagnaro et al., 2015) and to influence greenhouse gas emissions (Storer et al., 2018). While the role of AMF in biogeochemical cycles is undoubtedly complex, of pressing need is to determine the extent to which plants rely on these symbionts for mineral nutrient acquisition.

It is well established that AMF can contribute to plant N uptake (Ames et al., 1983; Hodge et al., 2001; Leigh et al., 2009; Thirkell et al., 2016), but the extent to which this takes place, and whether it is ecologically or agriculturally relevant is unclear (Smith and Smith, 2011a). This is in part due to relatively little experimental attention. There remains in the literature a focus on the role of AMF in plant phosphorus (P) uptake (Smith and Smith, 2011a; Karasawa et al., 2012; Ezawa and Saito, 2018), and consideration of symbiotic N uptake is often restricted to diazotrophic bacteria while AMF are often overlooked (Garcia et al., 2016).

Improved access to poorly-mobile soil P is, in most instances, the primary benefit of AMF to their plant hosts (Smith and Read, 2008). The relative immobility of inorganic P (Pi) in soil means that plant uptake of Pi from the rhizosphere can outpace Pi diffusion from the surrounding bulk soil and the subsequent P-depletion zones that form around the root are narrow and sharply defined. By engaging in symbiosis with AMF, with a mycelium spreading several centimetres beyond the rhizosphere, the plant effectively increases the volume of soil from which it can acquire nutrients, particularly poorly mobile ions such as Pi (Sanders and Tinker, 1973; Hodge, 2017). Nitrate (NO_3_^−^) and ammonium (NH_4_^+^), the predominant forms in which plants and fungi acquire N (Marschner, 2011), are more mobile in soil than orthophosphate (Tinker and Nye, 2000). Despite this, a zone of N-depletion may still form around the root (Brackin et al., 2017), in which case AMF may facilitate improved N capture for their plant hosts. With smaller diameters than plant roots, AMF hyphae may also penetrate soil micropores more effectively than a plant root, and thereby be present when inorganic N forms are released through microbial decomposition processes and effectively scavenge for this released inorganic N (Hodge, 2014).

Results from microcosm studies are conflicting as to the importance of AMF in plant N uptake (Hodge and Storer, 2015). While a number of studies have shown no improvement of N uptake by AM plants versus non-mycorrhizal counterparts (Cui and Caldwell, 1996a; Cui and Caldwell, 1996b; Reynolds et al., 2005; Kahkola et al., 2012), it is possible that AMF make an invisible contribution to nutrient acquisition which cannot easily be identified without the use of isotope tracing techniques. Mycorrhizal downregulation of plant root phosphate transporters has been identified in a number of studies (Smith et al., 2003; Smith et al., 2004). In this situation, AMF may be responsible for the majority of a plant’s P acquisition, but root transporter downregulation may result in reduced plant P uptake compared to non-mycorrhizal control plants (Smith et al., 2003; Smith et al., 2004). Whether a similar phenomenon occurs in mycorrhizal root N uptake remains unclear. Isotope tracing data does, however, show that AMF can transfer substantial amount of N to a host plant (Leigh et al., 2009; Thirkell et al., 2016), while the contribution of AMF to field-grown plant N uptake is unknown.

AMF are capable of acquiring N from decomposing organic sources (Leigh et al., 2009; Hodge and Fitter, 2010; Herman et al 2012; Barrett et al., 2014; Thirkell et al., 2016) and even to acquire some organic N directly from the hyphosphere, notably as amino acids (Hawkins et al., 2000; Breuninger et al., 2004; Whiteside et al., 2012a; Whiteside et al., 2012b, Tisserant et al., 2012) and perhaps as dipeptides (Belmondo et al., 2014). As in plants however, the vast majority of N acquired by AMF is thought to be as NO_3_^−^ or NH_4_^+^ (Govindarajulu et al., 2005; Bucking and Kafle, 2015). Greater N uptake as NO_3_^−^ might be expected as it is usually more abundant than NH_4_^+^ because of rapid nitrification (Marschner, 2011). However, because N acquired as NO_3_^−^ must be reduced to NH_4_^+^ before further assimilation, it should be energetically favourable for AMF to acquire N as NH_4_^+^ (Hodge et al., 2010; Courty et al., 2015). Corroborative data remains equivocal as to AMF “preference” for N types (Johansen et al., 1993; Hawkins and George, 2001). As NO_3_^−^ and NH_4_^+^ are the most commonly-used forms of fertiliser in Western agriculture, the need to understand mycorrhizal plant acquisition of these N sources is pressing.

Nutrient trade between partners in AM symbioses shows considerable variation in response to biotic factors such as plant and fungal genotype (Smith et al., 2004), in addition to abiotic factors including soil nutrient status (Johnson, 2010; Johnson et al., 2015). Despite substantial experimental data, predictability of the extent to which plants benefit from AMF colonisation remains poor. For example, no universally beneficial fungal isolate has been identified and comparatively few plants are obligate symbionts with AMF.

Despite the widespread distribution of AMF (Smith and Read, 2008; Davison et al., 2015) and the readiness with which they colonise most staple crop plant roots (Smith and Smith, 2011a), little is understood about the function of AMF in the field (Lekberg and Helgason, 2018; Ryan and Graham, 2018). Most published material on the function of AMF is derived from studies conducted under controlled conditions, often comparing AM plants with non-AM controls. While such experiments have provided much valuable data and insight, their findings cannot directly be extrapolated to the field scale, as the occurrence of non-AM cereals in most arable soils is unlikely (Smith and Smith, 2011a). Despite disruptive practices such as tilling and the application of fungicides, there remains a substantial AMF spore bank (and therefore inoculum potential) in agricultural soils (Sosa-Hernandez et al., 2018) and it is very likely that plants in arable field soil will be colonised by AMF (Smith and Smith, 2011a). Further research is needed to begin to understand how AMF might affect crop plant nutrient uptake *in situ*.

Adding ^15^N isotope tracers to mesh-walled soil compartments in a field trial, we examined the role of AMF in the N acquisition by barley (*Hordeum vulgare* L.) cultivars “Meridian” and “Maris Otter”. Isotopic ^15^N labelling was carried out in plots receiving contrasting N rates to test the impact of N availability on nutrient transfer in the symbiosis. We tested the hypothesis that increased N fertilisation would result in more AMF transfer of N to host plants because AMF, by virtue of their size, would be better able than roots to compete with the soil microbiome for the added N held in physically small microsites. N tracers were added as NH_4_^+^ or NO_3_^−^ to investigate the relative uptake and transfer of different N sources by AMF.

## Materials and Methods

### Field Trial Design

Data were gathered from a larger field trial, designed and implemented at Sancton, East Riding of Yorkshire (co-ordinates 53°51′10.2″N 0°35′29.1″W), by ADAS (Pendeford, Wolverhampton, UK). The ADAS trial was set up to test how barley yield compares among 6 application rates of ammonium nitrate (NH_4_NO_3_) fertiliser (Nitram, CF Fertiliser, Ince, Cheshire, UK) ranging from 0–300 kg ha^−1^. The soil at the trial site comprises a silty rendzina, with a significant proportion of chalk fragments (UKSO, 2016). Soil mineral N, quantified shortly before sowing, was 29.9 kg N ha^−1^, of which 28 kg was nitrate-N and 1.9 kg ammonium-N. The field site on which the trial was based is a commercial arable farm, with barley (*Hordeum vulgare* L.), oilseed rape (*Brassica napus* L.) and wheat (*Triticum aestivum* L.) grown in a rotation.

**Figure 1:**
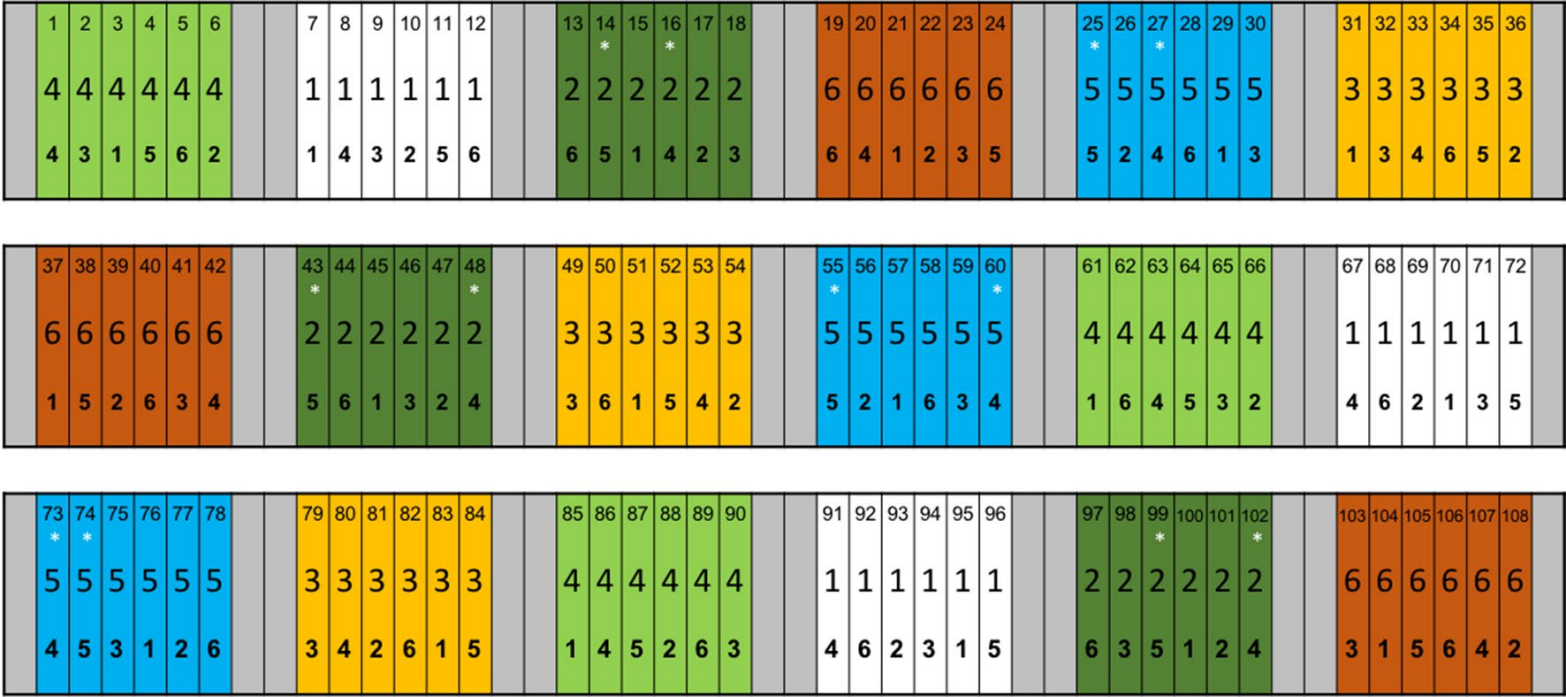
ADAS experiment established at Sancton, East Riding of Yorkshire, UK. Six barley (*Hordeum vulgare* L.) cultivars were planted at the trial site, and received one of 6 N addition rates, ranging from 0 to 300 kg ha^−1^. Each combination of barley cultivar and N rate was replicated 3 times. Each plot has 3 numbers, denoting: plot identity, N addition rate and barley cultivar, reading top to bottom. Nitrogen addition rate “2” represents 60 kg ha^−1^ and “5” is 280 kg ha^−1^. Plot colours also represent N addition rate. Meridian barley is denoted by “4” and Maris Otter by “5”. Asterisks (*) represent plots from which root samples were taken for analysis of root length colonisation and to which ^15^N tracer was added. Reproduced with permission from Pete Berry and Kate Storer, ADAS.

The ADAS trial used plots measuring 12 m × 1.5 m, clustered in groups of 6 by N application rate, with each variety represented once per cluster. Each N application rate was applied to 3 replicate clusters, of 6 varieties, meaning 18 clusters in total, with a combined area of 1944 m^2^. Experimental clusters of N application rates were separated to each side by buffer zones 6 m wide, and at each end by buffer zones 3 m long (**Figure 1**). Owing to the logistical challenges of sampling the entire trial, the experimental work presented here is gathered from two of the N application rates (60 kg ha^−1^ (N rate 2 in **Figure 1**), and 280 kg ha^−1^ (N rate 5 in **Figure 1**)), and two of the barley cultivars: KWS Meridian (KWS UK Ltd, Thriplow, Hertfordshire, UK), a 6-row feedstock barley; and Maris Otter (Robin Appel, Waltham Chase, Hampshire, UK), a 2-row malting barley, giving 4 treatment groups, with 3 replicate plots per treatment. Meridian and Maris Otter were chosen from the panel of 6 cultivars available in the trial as they represent contrasting ages of barley varieties, developed in the 1960s and 2000s respectively. Further, Maris Otter is a malting barley, characterised by a low grain protein content, while Meridian was developed as a feedstock barley, with a higher grain protein (and therefore N) content. Experimental sampling and isotope labelling were carried out during the post-anthesis, grain filling period — approximate growth stages 70–80 (Zadoks et al., 1974).

### Intraradical and Extraradical AMF Quantification

AMF colonisation of both barley varieties was confirmed and then quantified by staining of roots collected from the trial plots. Roots were collected from between 5 and 15 cm below the surface. After clearing in 10% (w/v) KOH for 20 min at 70°C, roots were rinsed in de-ionised water, acidified in 1% (v/v) HCl at 25°C for 10 min and then stained in Trypan Blue at 25°C for 20 min. Roots were then rinsed again in de-ionised water before being left in a 50% (v/v) glycerol solution for 24 h, before being mounted onto microscope slides to allow quantification of root length colonisation (RLC) using the gridline intersect method (McGonigle et al., 1990).

Soil samples were collected from between 5 and 15 cm below the soil surface. As AMF hyphal turnover can be rapid (Staddon et al., 2003), hyphal extraction took place within 6 h of collection to minimise loss due to decomposition. Extraradical hyphal quantity in the plots was determined using an adapted method from Staddon et al. (1999). Briefly, samples of known mass (5–10 g) were suspended in 500 mL of de-ionised water and agitated with a magnetic stirrer plate in order to free the hyphae from soil particles. From this, 200 mL was decanted to a smaller beaker on a magnetic stirrer. Aliquots (10 mL) were removed and vacuum filtered through 0.45 µm nylon mesh (Anachem, Bedfordshire, UK) and hyphal length density (HLD) was quantified using the gridline intersect method (Hodge, 2001).

### ^15^N Stable Isotope Labelling

The AMF contribution to barley N uptake was investigated by adding a solution of ^15^N (as either (^15^NH_4_)_2_SO_4_ or K^15^NO_3_), into mesh-walled cores, into which AMF hyphae could access but plant roots could not, or (as controls for diffusion and mass flow of the added N) cores into which neither AMF hyphae or roots could access. Isotopic ^15^N was added in the form of Long Ashton nutrient solution (LAS) (Smith et al., 1983), which can be prepared variously to provide ^15^N as ^15^NH_4_^+^ or ^15^NO_3_^−^ in equimolar concentrations. The LAS was made to the standard protocols except N being 300% the original concentrations. Each core received 5 mL of LAS, containing 0.683 mg ^15^N. (Long Ashton nutrient solution protocol is included in **Supplementary Information Document 1**).

Hyphal access cores were constructed following an adapted method from Johnson et al. (2001). Lengths of PVC tubing (length 85 mm, internal diameter 13 mm, external diameter 16 mm; internal volume 9.9 cm^3^) with 2 windows cut in the sides of the lower ^2^/_3_ of the tube so that 50% of the side area was open, were wrapped in a 20 µm nylon mesh (John Stanier and Co., Whitefield, Manchester, UK), fixed with Tensol adhesive cement (Bostik Inc., Wauwatosa, Wisconsin, USA). The open bottom end of each tube was covered with the same size mesh. Control cores, which allowed diffusion and mass flow of solutes but prevent hyphal ingrowth, were covered with 0.45 µm nitrocellulose membrane mesh to prevent root and hyphal ingrowth. Cores were filled with a 1/1 (v/v) mixture of silica sand and TerraGreen^®^ (calcinated attapulgite clay, Oil-Dri, Cambridgeshire, UK), which had been sterilised by autoclaving (121°C for 44 min), providing a uniform substrate into which the ^15^N solutions could be added.

Each of these cores was then placed inside another, slightly larger core, constructed in the same manner (length 75 mm internal diameter 18 mm, external diameter 21 mm). These cores were also covered in a 20 µm nylon mesh. Such a “core in a core” design allows the placement of zones of defined and uniform size into the soil, to which ^15^N label solutions could be added. A small (approximately 1 mm) air gap is made between the external mesh wall of one core and the internal mesh wall of the other, which should reduce the rapid diffusion of N from the site of addition, which has been a problem in studies where ^15^N has been added (Smith and Smith, 2011b). Diffusion and mass flow are unlikely to be prevented entirely, as the pressure of soil on the sides of the core may push the mesh together so that the two layers of mesh make contact. However, the system provides a more stable labelling zone than using a single core, where one mesh layer may be easily damaged (Johnson et al., 2001).

**Figure 2:**
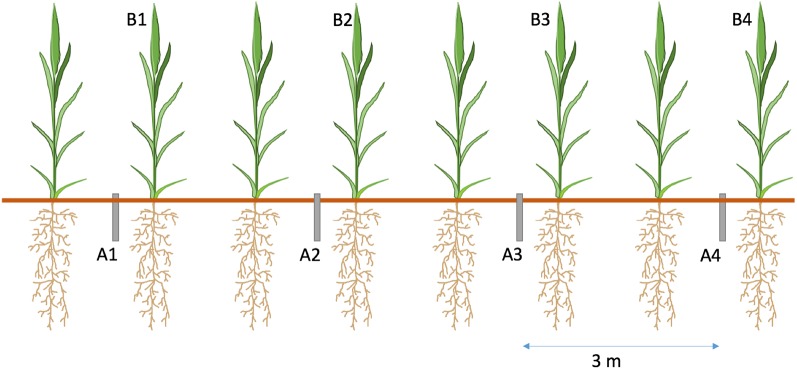
Diagram of ^15^N addition experiment. PVC cores were inserted adjacent to barley (*Hordeum vulgare* L.) plants, four cores per plot, spaced 3 m apart. Cores were organised as follows A1 – AMF Access + Ammonium (NH_4_^+^); A2 - No AMF Access + Nitrate (NO_3_^−^); A3 - AMF Access + NO_3_^−^; A4 - No AMF Access + NH_4_^+^ Each core received 0.683 mg ^15^N added as Long Ashtons nutrient solution. Plant shoots closest to the core (B1-4) were removed, dried and homogenised for N analysis. B1-B4 denote shoot samples taken.

Each of the 12 experimental plots received four cores (1. No AMF Access + ^15^NH_4_^+^; 2. AMF Access + ^15^NH_4_^+^; 3. No AMF Access + ^15^NO_3_^−^; 4. AMF Access + ^15^NO_3_^−^), spaced 3 m apart to avoid contamination of ^15^N from neighbouring cores (**Figure 2**). Placement of cores took place 8 weeks before label addition to allow hyphal ingrowth from the bulk soil. A piece of tape was placed over the top of cores to minimise contamination. This tape was removed for ^15^N addition and then replaced.

### Sample Collection and Preparation

After 7 days, the nearest plant to each labelling core was cut at ground level and removed, dried at 70°C for 48 h and homogenised in a kitchen blender (Morphy Richards, Mexborough, South Yorkshire, UK) then in a ball mill (MM400 Ball Mill, Retsch GmbH, Haan, Germany). Homogenised shoot samples of known mass (3 mg ± 0.5 mg) were used to quantify ^15^N and N content, performed by isotope ratio mass spectrometry (IRMS) (PDZ 2020, Sercon Ltd, Crewe, UK).

### Statistical Analysis

For all data, statistical analysis was performed using the “R 3.1.0” statistical package, through the “RStudio” integrated development environment (R foundation for Statistical Computing, Vienna, Austria). Data were tested for normality using Shapiro-Wilk and Kolmogorov-Smirnov tests, and Levene’s test was used to confirm homogeneity of variance. Where these tests suggested data did not match test assumptions, data were square-root or log-transformed prior to analysis. Data for root length colonisation, hyphal length density, barley N concentration and biomass were tested by two-way ANOVA, using N addition rate and barley variety as explanatory variables. As two additional explanatory variables were added in the trial for ^15^N uptake (N addition type, ammonium/nitrate; AM treatment, access/no access), and the small number of replicates in the ADAS field trial, it was not possible to test these factors and the N addition rate and barley cultivar at once. As such, data were split into barley cultivar and N application rate for the ^15^N data and tested by two-way ANOVA. Here, ^15^N enrichment was the response variable, while N type and AMF access treatment were the explanatory variables.

## Results

**Figure 3:**
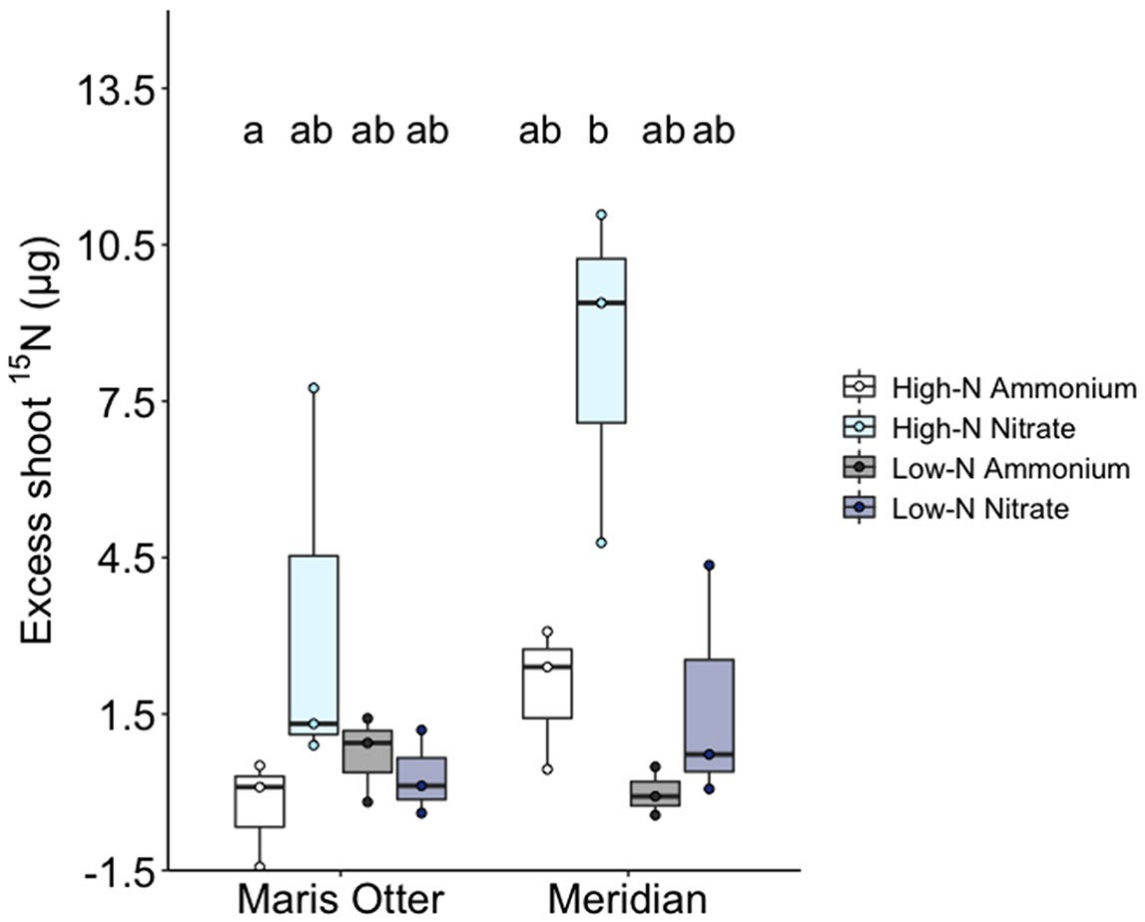
Excess ^15^N content in Maris Otter and Meridian shoots (calculated by subtracting shoot ^15^N content in each “Access” unit from the mean of the corresponding values in the “No Access” units). Shoot ^15^N enrichment was significantly higher than “no access” controls when supplied as nitrate to Meridian barley in High-N plots. Circles represent individual data points, boxplot centre bars represent the median values. High-N + ammonium groups are represented by white bars, High-N + nitrate by light blue bars, Low-N + ammonium by dark grey bars and Low-N + nitrate by dark blue bars. Data shown are means ± SEM, n = 3. Bars sharing the same letter are not significantly different.

Shoot acquisition of ^15^N added to mesh cores was significantly improved by allowing AMF access into cores, but only when added as ^15^NO_3_^−^, and only in the High-N plots of Meridian barley (**Figure 3**). T-tests indicate that only in High-N Meridian plots receiving ^15^NO_3_^−^ were ^15^N enrichment levels greater in the AM access treatment than in the no access controls (*T* *_2_* = 4.48, *p = 0.023*)(**Supplementary Information, Figure 1**). A two-way ANOVA showed that in High-N Meridian, there was a significant effect of N source (*F*_1,8_ = 12.73, *p* = 0.007) and AMF access to cores (*F*_1,8_ = 27.86, *p* = 0.007). There was also a significant interaction between N source and AMF access (*F*_1,8_ = 14.25, *p* = 0.005) (**Figure 3**). In High-N Meridian with AMF access, the harvested plants, i.e. those individuals closest to the core to which the isotope label was added, acquired on average 1.62% of the ^15^N supplied. Other treatment groups saw no greater plant uptake of ^15^N where AMF could access the isotope label than in no-access controls. Excepting High-N Meridian plots, mean shoot ^15^N content did not differ among treatments and controls, indicating similar plant acquisition of N following diffusion/mass flow out and into the soil, but minimal fungal-mediated uptake.

**Figure 4:**
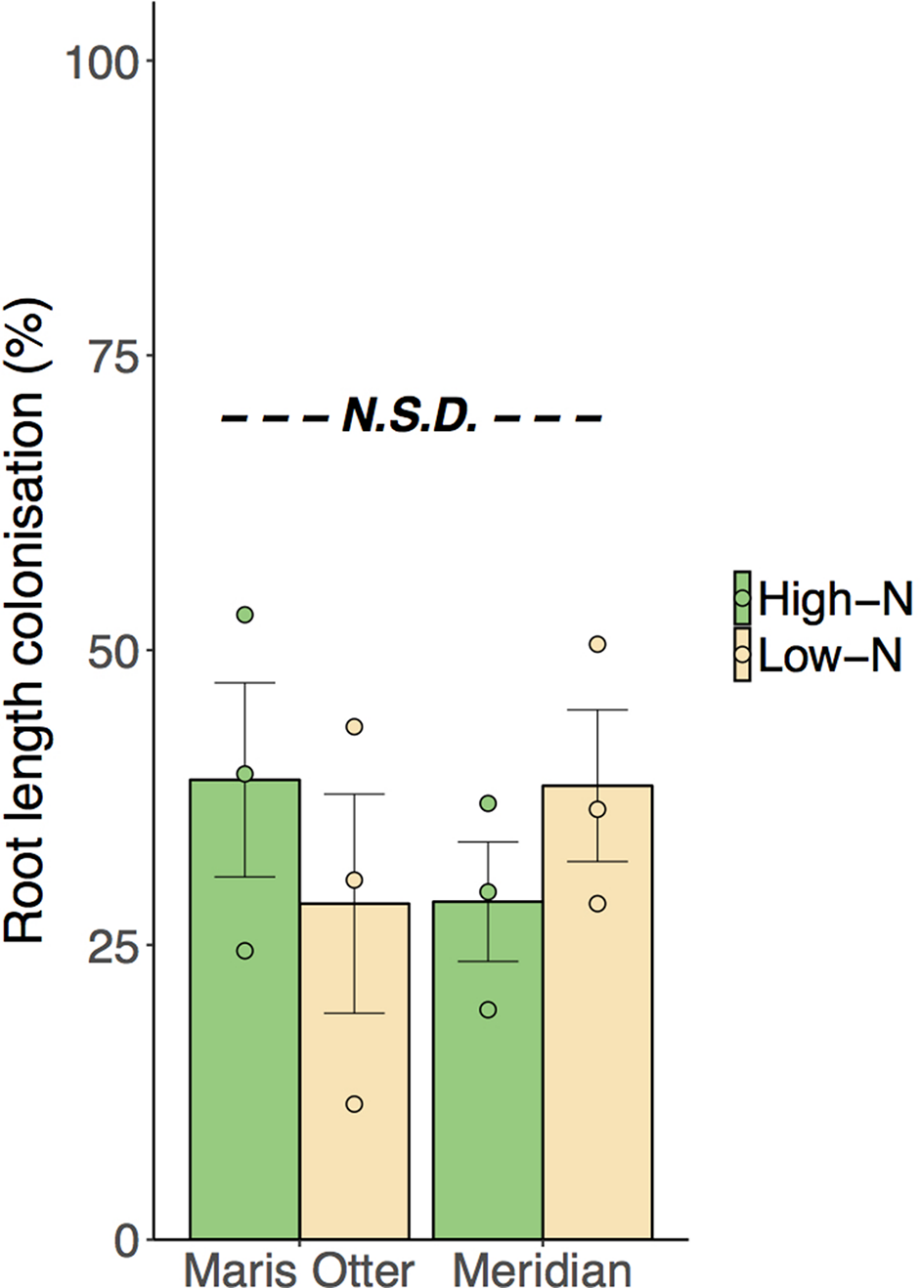
Percentage root length colonisation, as determined by Trypan Blue staining, was not significantly different between treatments. All inspected plants were colonised by arbuscular mycorrhizal fungi (AMF), confirmed by presence of characteristic structures, arbuscules and vesicles. Mean colonisation ranged from 28.5% in Maris Otter in Low N, to 38.0% in Meridian Low-N, but no groups were significantly different. Circles represent individual data points. High-N groups are denoted by green bars, Low-N groups are denoted by yellow bars. “N.S.D.” indicates that there were no significant differences among treatment means. Data shown are means ± SEM, n = 3.

All plant roots studied were found to be colonised by AMF, indicating a substantial inoculum potential of the soil at the trial site, although no differences were found between cultivar or N-rate treatments (*p* > 0.05). Mean colonisation was 33.7% (± 3.52% SEM) across all treatment groups (**Figure 4**). Extraradical mycelium (ERM) hyphal densities, measured in the zones to which ^15^N was added, were not different among treatment groups (*p* > 0.05). Mean ERM hyphal density across all treatments was 2.49 m g^−1^ DW soil (± 0.31 m g^−1^ SEM). In both cultivars, High-N plots supported ∼ 60% higher shoot N content than Low-N plots (*F*_1,8_ = 74.55, *p* < 0.001), and shoot N concentration was significantly higher in High-N than Low-N plots (*F*_1,8_ = 84.28, *p* < 0.001). Mean shoot N concentration was 9.30 mg g^−1^ DW in Low-N blocks of Maris Otter, and 14.75 mg g^−1^ DW in the High-N. Meridian showed a very similar trend, as N concentration increased from 9.57 mg g^−1^ DW in Low-N plots to 14.38 mg g^−1^ DW in the High N. Shoot N concentration and content did not differ between the two cultivars tested. Shoot DW did not differ between the varieties or the N addition rates.

## Discussion

The enrichment of ^15^N in barley shoots suggests a role for AMF-facilitated N acquisition by crop plants, a phenomenon not previously observed in a field setting. Moreover, our data suggest this route of N uptake is dependent upon barley cultivar identity, the N form added and the rate at which N has previously been applied to the plots. AMF have been shown to transfer substantial quantities of N to plants in root organ culture experiments (Jin et al., 2005) although caution must be exercised before extrapolating these values to crop plant systems as they are far-removed from realistic mycorrhizal physiology. Whole-plant microcosm studies conducted under greenhouse conditions have given mixed results as to whether AMF may contribute to plant N nutrition (Hodge and Storer, 2015). Our data provide the first suggestion that AMF may have a role in cereal crop N uptake in the field. Our data also suggest that short-term changes in N fertilisation regimes can elicit shifts in AM functioning.

While our data suggest a preference for AMF to transfer N to plants when provided to this system as NO_3_^−^ rather than NH_4_^+^, previous experimental evidence as to inorganic N source preference by AMF is equivocal (Johansen et al., 1993; Hawkins and George, 2001). Higher uptake of NO_3_^−^ than NH_4_^+^ is contrary to models which suggest NH_4_^+^ acquisition should be less energetically expensive (Govindarajulu et al., 2005). Hyphal NH_4_^+^ uptake may be retarded by problems of charge balancing that are perhaps not encountered when N is acquired as NO_3_^−^. Simultaneous uptake of NO_3_^−^ and cations such as K^+^, Ca^2+^ or Mg^2+^ from the soil may avoid changes in electrochemical potential across exchange surfaces, allowing N acquisition. Meanwhile, NH_4_^+^ uptake would require proton secretion (or anion uptake), which may shift soil pH making further NH_4_^+^ uptake more difficult. Nitrate-N comprised over 90% of the available N in the soil before the trial was planted, a trend which is not unusual, as NO_3_^−^ often dominates inorganic N pools in arable soils (Marschner, 2011). These relative abundances of N sources may have led to AMF hyphal physiology being acclimated to nitrate uptake (Garraway and Evans, 1984), meaning suddenly-available NH_4_^+^ could not be acquired effectively. Although the movement and cycling of nitrate and ammonium are known to be influenced by soil moisture (Homyak et al., 2017), precipitation data for the site (**Supplementary Information, Table 1**) indicates no extraordinary rainfall in the weeks over which the experiment took place, suggesting this was of minor importance here.

While recovery of only 1.6% of the ^15^N label seems low, total ^15^N recovery is likely to have been greater than the data suggests. Our data are derived from the aboveground tissue of one plant proximal to the mesh-walled core into which isotopes were added, and it is probable that the roots of numerous plants would have been in close proximity to the core. As such, further ^15^N is likely to have been acquired by multiple plants. Furthermore, greater ^15^N uptake into plant shoots may have been recorded if the shoot tissue samples had been taken longer after ^15^N addition to the mesh-walled cores.

Mesh-walled exclusion cores have been used to quantify AMF-plant nutrient dynamics in a number of studies (Johnson et al., 2001; Field et al., 2012; Field et al., 2016), and are of particular utility where the establishment of truly non-mycorrhizal control plants is not feasible, as in this study. The use of a 0.45 µm nitrocellulose membrane to exclude AMF in-growth to soil compartments is a well-established methodology in the literature (Hodge et al., 2001; Leigh et al., 2009; Thirkell et al., 2016; Storer et al., 2018), although some concerns arise in relation to the effects of such small pore sizes on solute movement, although in the case of studies investigating mycorrhizal P uptake, such effects have been determined to be insignificant (see Zhang et al., 2016; Svenningsen et al., 2018). Our data show increased plant ^15^N uptake in plots only where N was supplied as nitrate, to Meridian barley, and in plots which had received high rates of N fertiliser (**Figure 3**). Were the movement of N through these systems determined by the porosity of the membranes used in “no access” treatments, we might expect ^15^N enrichment in all plots which received ^15^NO_3_^−^, which is not the case. Alternative control treatments to disentangle the effects of AMF on plant nutrition might be tested further in future studies to determine the relative merits of each method. Non-mycorrhiza-forming mutants of a number of cereals have been developed (Paszkowski et al., 2006; Watts-Williams and Cavagnaro, 2015) but to date no mycorrhizal-defective barley mutants are available against which data from hyphal exclusion experiments can be compared. Furthermore, an AMF-colonised plant is morphologically (Gutjahr et al., 2009) and physiologically (Luginbuehl and Oldroyd, 2017) distinct from one which remains uncolonised, and comparisons between AM and mycorrhiza-defective mutants may erroneously conflate these differences and ascribe all contrasts to the lack of mycorrhizas. Combinations of experimental approaches may be employed here to improve the rigour of field experimentation, although the logistics of such trials may prove represent a significant challenge.

Identifying the mechanisms responsible for differential nitrogen transfer from fungus to plant are beyond the scope of this study, but a number of possibilities may be considered. Numerous studies have demonstrated shifts in AMF community composition or structure following N fertilisation, in grassland (Egerton-Warburton and Allen, 2000; Egerton-Warburton et al., 2007; Antoninka et al., 2011; Jiang et al., 2018) and arable systems (Verbruggen et al., 2010; Avio et al., 2013; Liu et al., 2014; Williams et al., 2017). As AMF isolates are known to be functionally different (Avio et al., 2006; Mensah et al., 2015) any N-driven shifts in AMF community have the potential to influence the N cycling in the system (Herman et al., 2012). Future experimental testing of the AMF community composition within cereal roots, combined with isotopic tracer studies may elucidate any link between the structure and function of AMF communities in agronomic systems.

## Conclusions

Our data show that AMF transfer of N to plant hosts is influenced by agricultural management decisions, here the cultivar of barley and the rate at which inorganic N fertiliser is supplied. The extent to which symbiotic soil microbes might enhance total nutrient uptake in the field remains to be tested; despite demonstrating a mechanism by which plants acquire N, our data cannot indicate whether non-AMF plants in the same field conditions might show enhanced nutrition. Further experimental investigation is required for a wider perspective on the influence of these fungi on their crop plant hosts, and therefore their importance in agricultural systems.

## Data Availability Statement

The datasets generated for this study are included within the supplementary information of this manuscript.

## Author Contributions

TT, DC, and AH designed the study. TT carried out experimental work and data analysis and wrote the initial draft of the manuscript. All authors contributed to revisions of the manuscript, and read and approved the final submitted version.

## Funding

This work was supported by a BBSRC White Rose DTP grant: BB/J014443/1.

## Conflict of Interest

The authors declare that the research was conducted in the absence of any commercial or financial relationships that could be construed as a potential conflict of interest.
