# Supplementary material for: Contrasting Nitrogen Fertilisation Rates Alter Mycorrhizal Contribution to Barley Nutrition in a Field Trial
Source: Front Plant Sci. 2019 Oct 30;10:1312. doi: 10.3389/fpls.2019.01312 (PMC6831614; doi:10.3389/fpls.2019.01312)
Supplement: Supplementary file 1 [file Image_1.pdf]

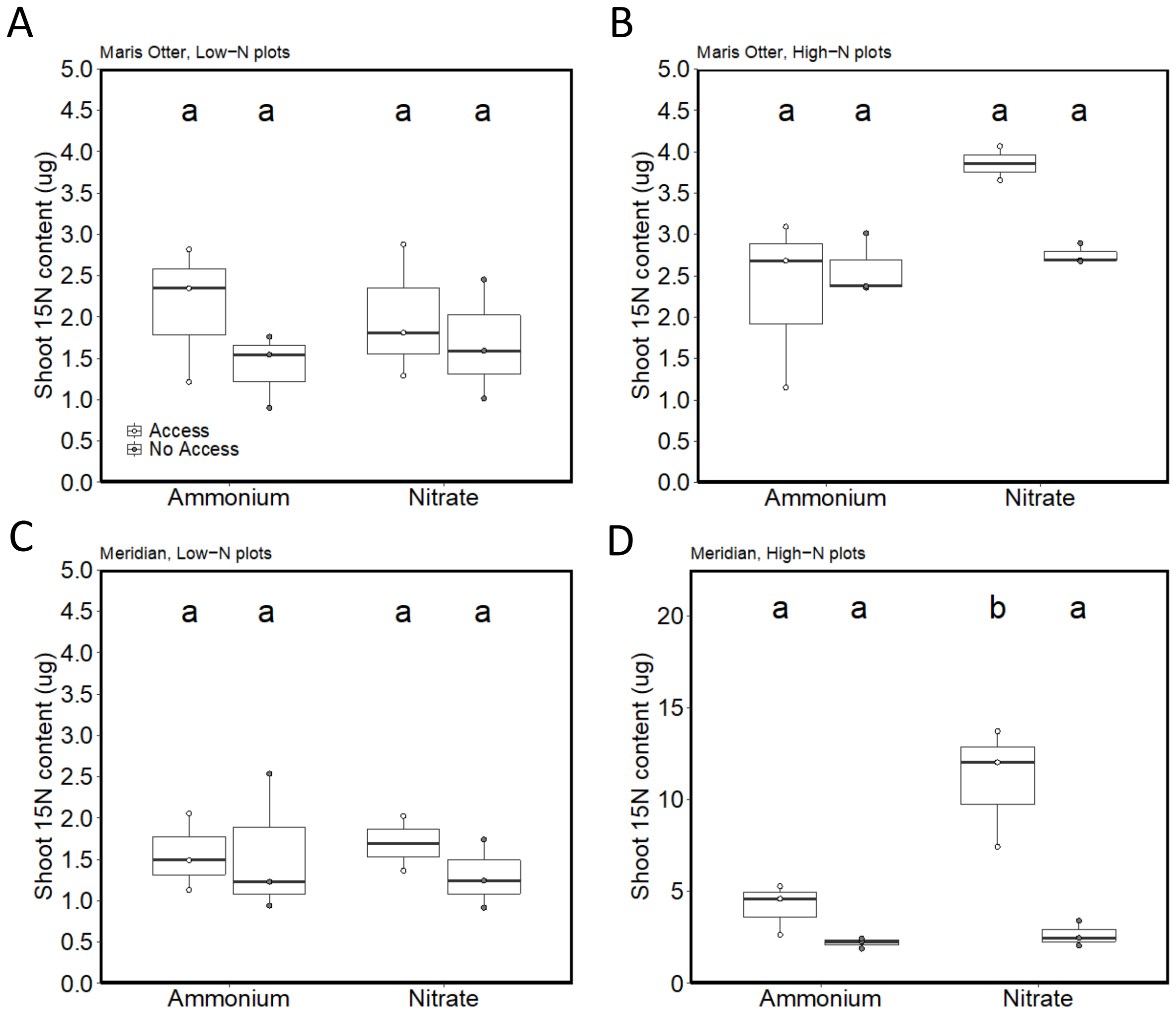

Supplementary Figure 1. ^{15}N excess in aboveground tissue of Maris Otter (A,B) and Meridian (C,D) barley under high (280 kg Ha^{-1}) and low (60 kg Ha^{-1}) nitrogen fertilisation rates, where ^{15}N is added as nitrate (NO_3^-)or ammonium (NH_4^+). White boxes represent the 'mycorrhizal access' treatment and grey boxes the 'no mycorrhizal access' treatment, where isotopic ^{15}N labels were added to cores with 20 µm mesh walls, or 0.45 µm mesh walls, respectively.

Long Ashton nutrient solution preparation — Nitrate formulation

Compound	Concentration in solution (mM)
$K^{15}NO_3$	12
$Ca(NO_3)_2.4H_2O$	4
$NaH_2PO_4.2H_2O$	1.17
$MgSO_4.7H_2O$	1.5
FeNaEDTA	0.09
$MnSO_4.4H_2O$	0.01
$ZnSO_47H_2O$	0.001
$CuSO_4.5H_2O$	0.001
H_3BO_3	0.05
$NaMoO_4.2H_2O$	0.0005
NaCl	0.1

Long Ashton nutrient solution preparation — Ammonium formulation

Compound	Concentration in solution (mM)
$(^{15}NH_4)_2SO_4$	12
K_2SO_4	1.9
$CaCl_2.2H_2O$	4
$NaHPO_4.12H_2O$	1.28
$MgSO_4.7H_2O$	1.5
FeNaEDTA	0.09
$MnSO_4.4H_2O$	0.01
$ZnSO_47H_2O$	0.001
$CuSO_4.5H_2O$	0.001
H_3BO_3	0.05
$NaMoO_4.2H_2O$	0.0005
NaCl	0.1
